# Supplementary figures and images for: Telomere-to-telomere genome assembly of an allotetraploid pernicious weed, Echinochloa phyllopogon
Source: DNA Res. 2023 Nov 7;30(5):dsad023. doi: 10.1093/dnares/dsad023 (PMC10634394; doi:10.1093/dnares/dsad023)

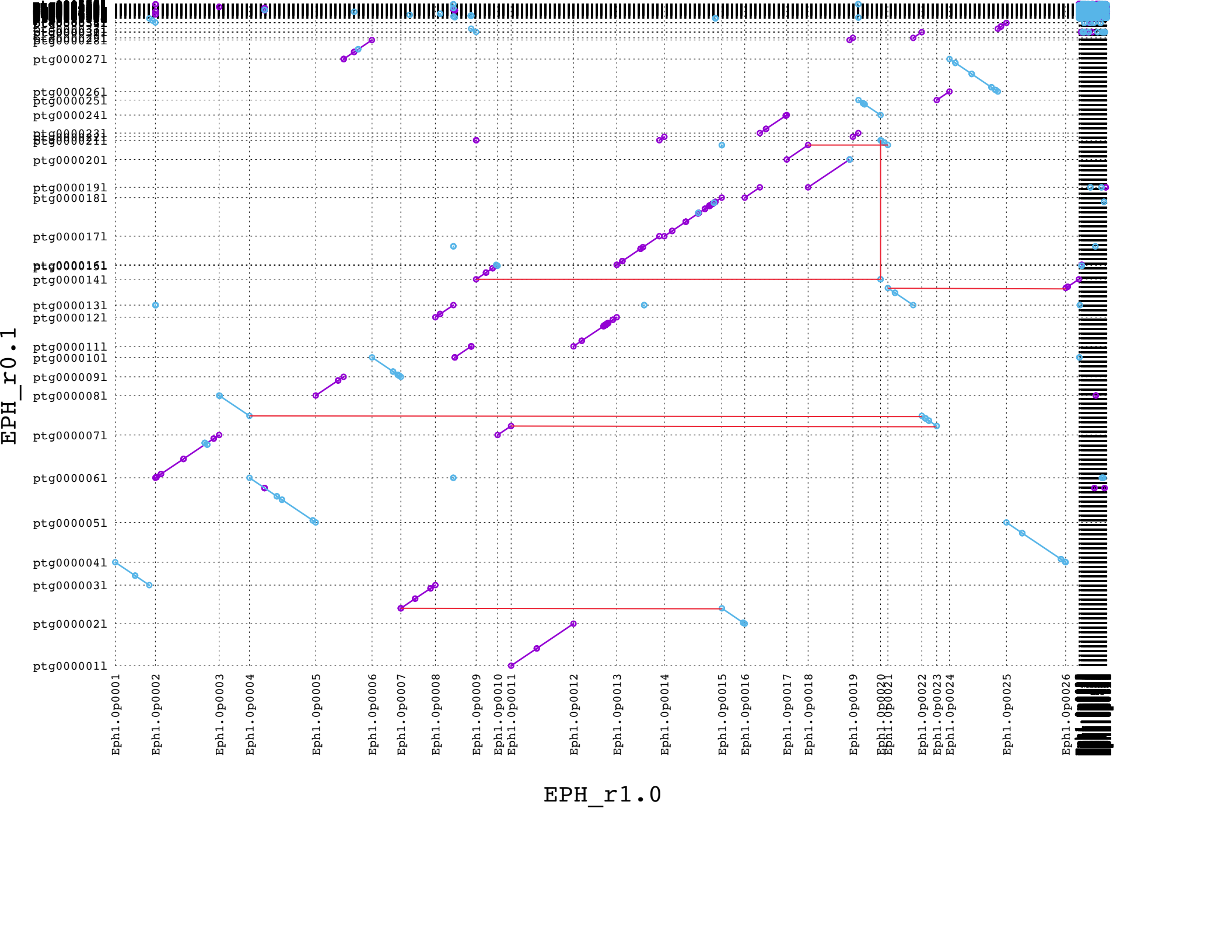

Supplement: dsad023_suppl_Supplementary_Figures_S1 [file dsad023_suppl_supplementary_figures_s1.pdf]

**A**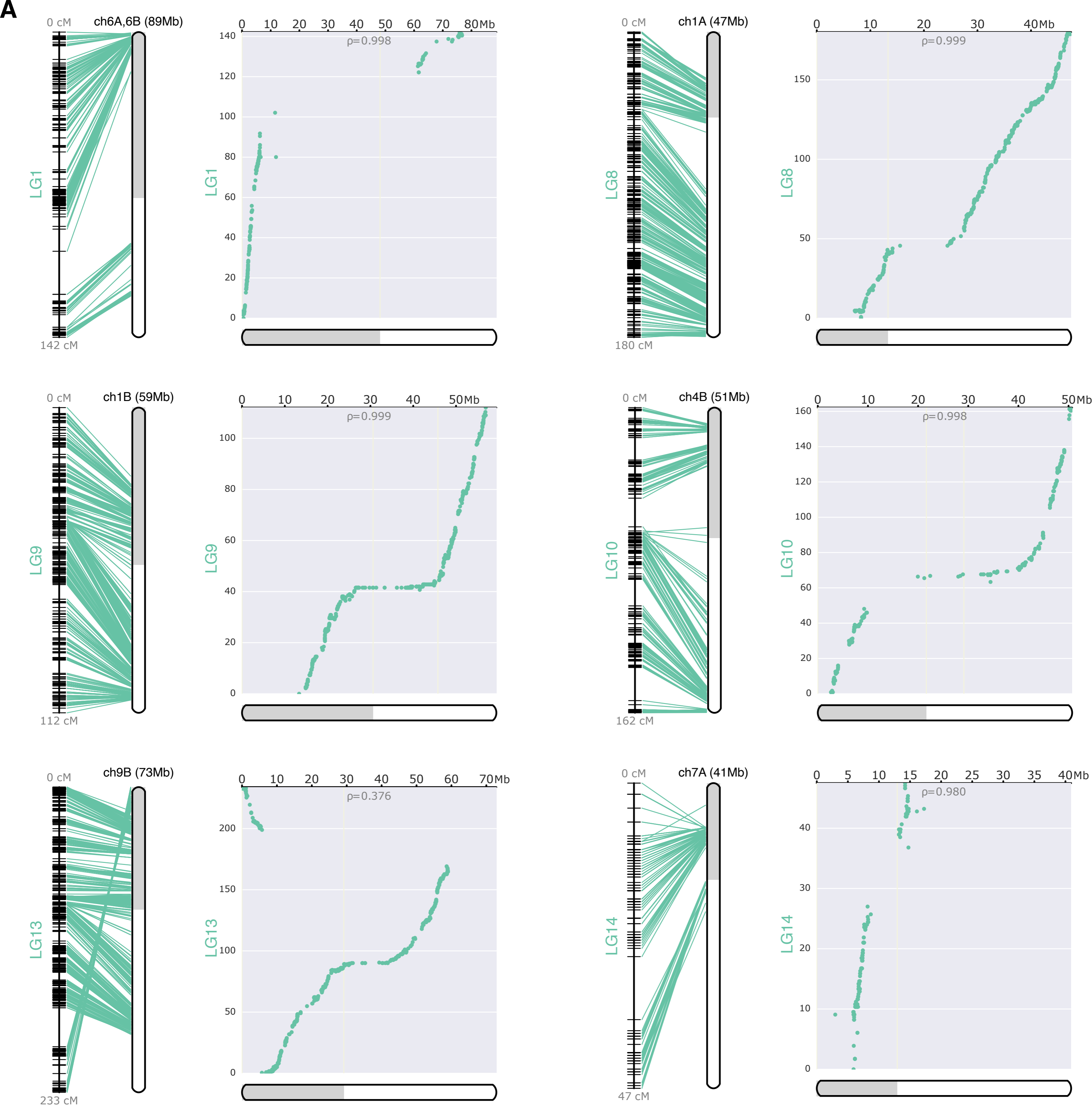**B**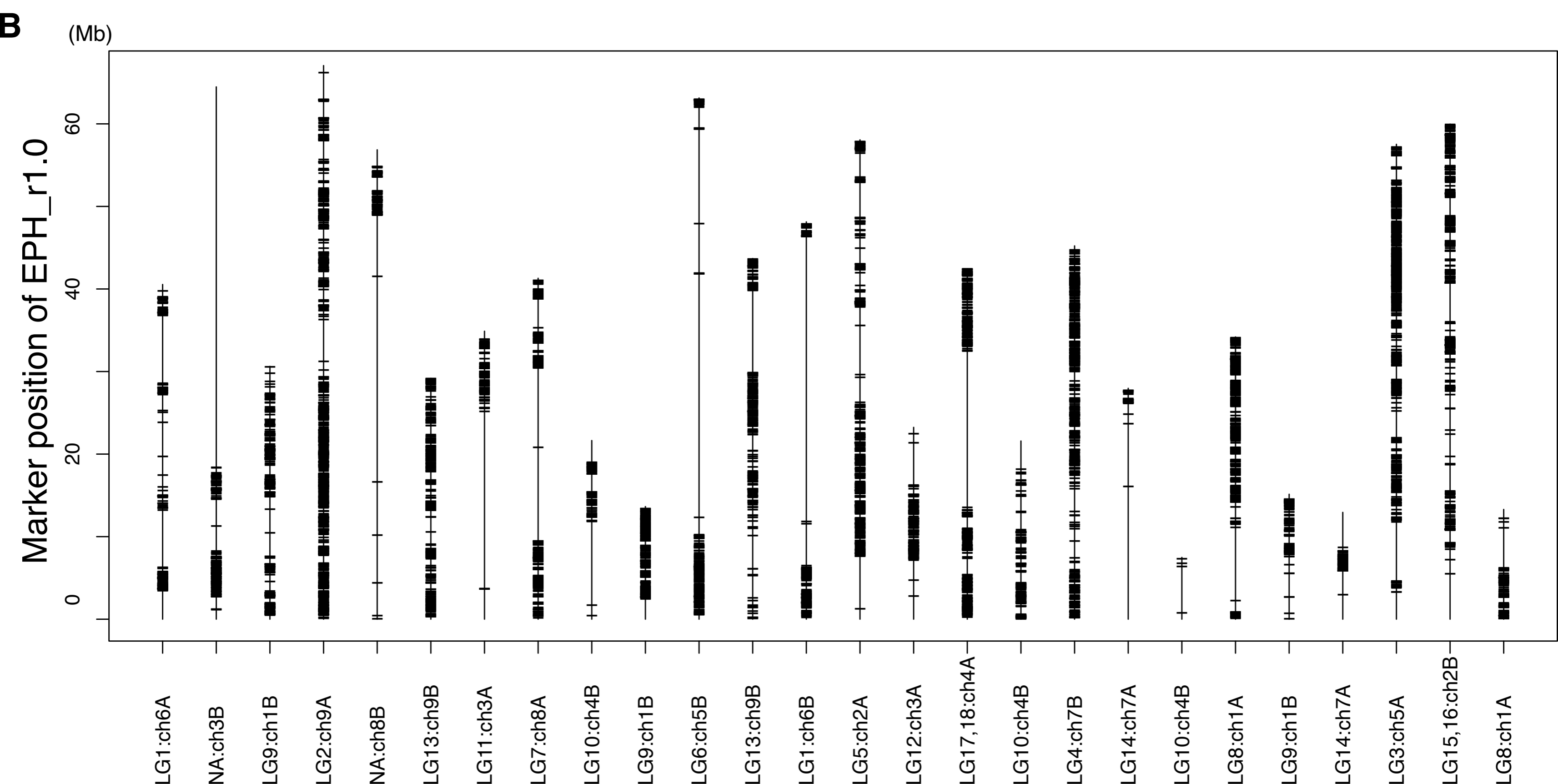

Supplement: dsad023_suppl_Supplementary_Figures_S2 [file dsad023_suppl_supplementary_figures_s2.pdf]

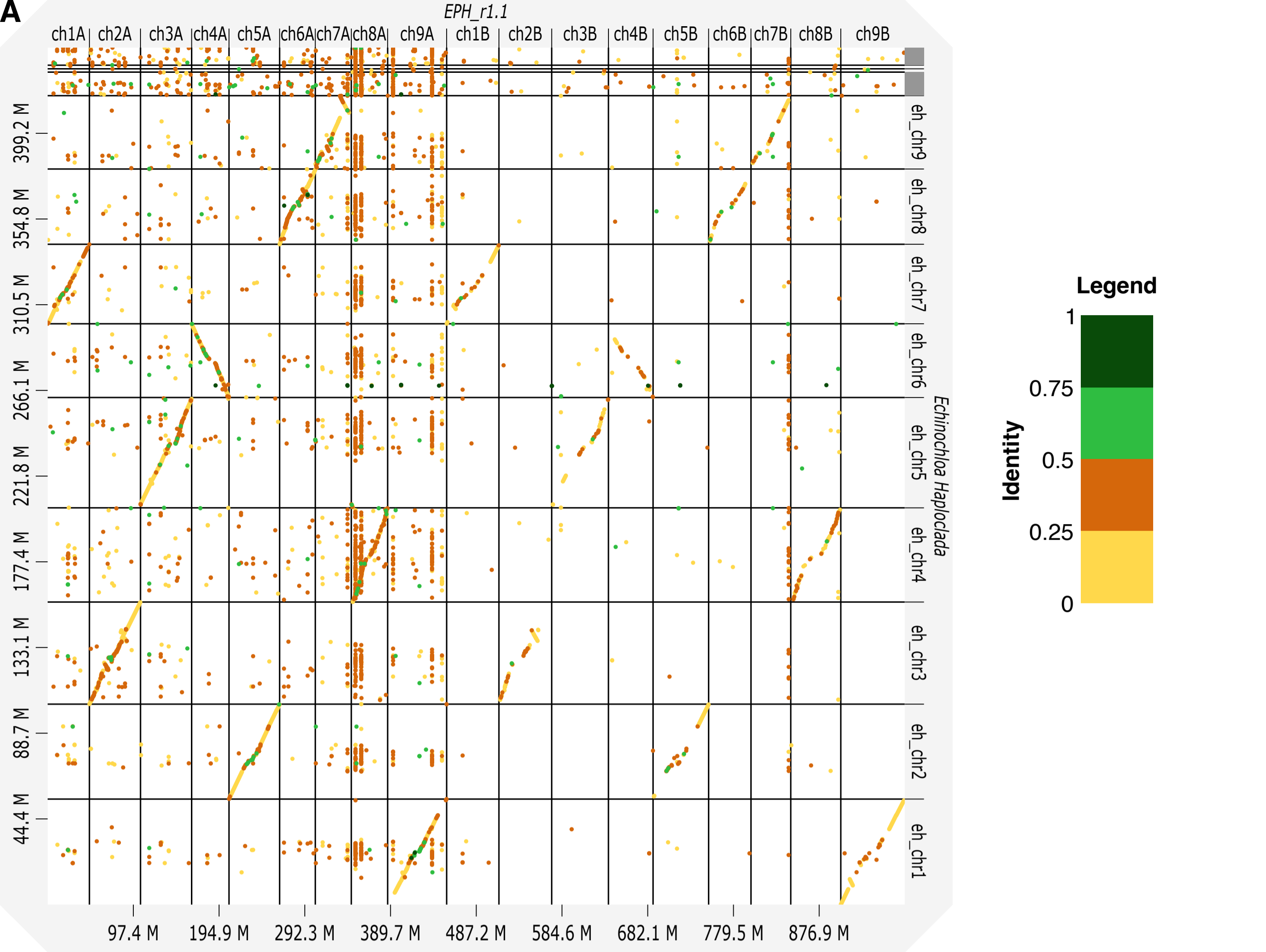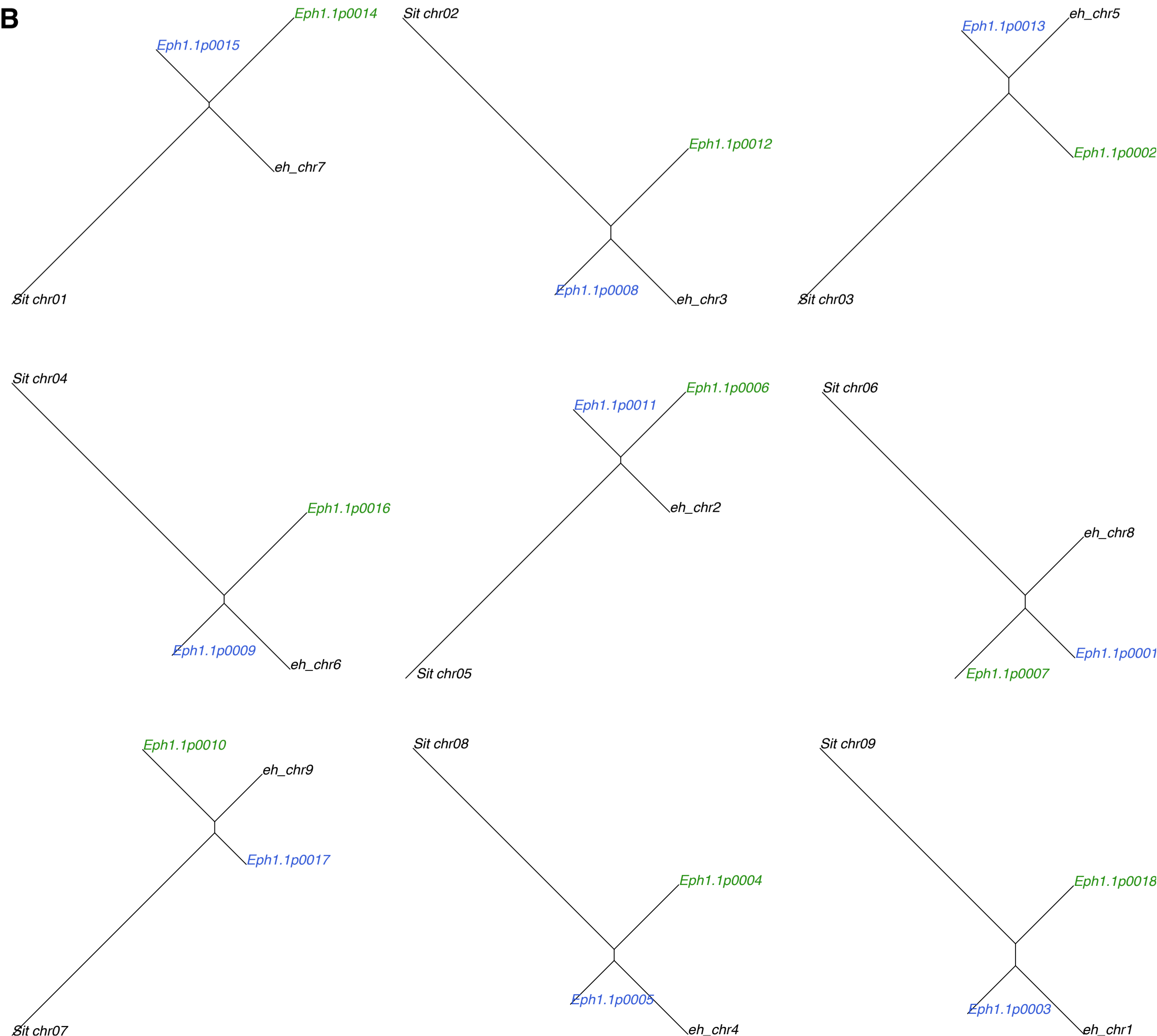

Supplement: dsad023_suppl_Supplementary_Figures_S3 [file dsad023_suppl_supplementary_figures_s3.pdf]

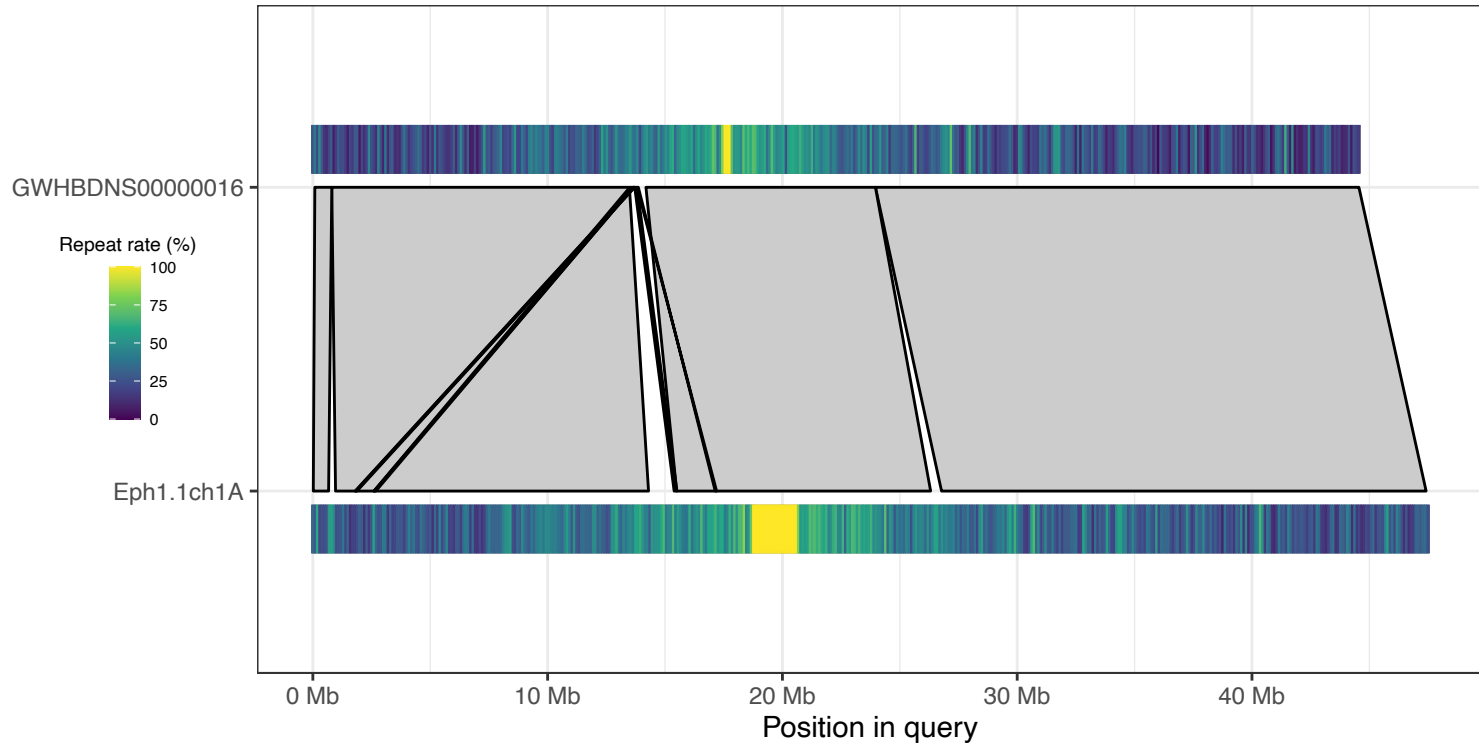

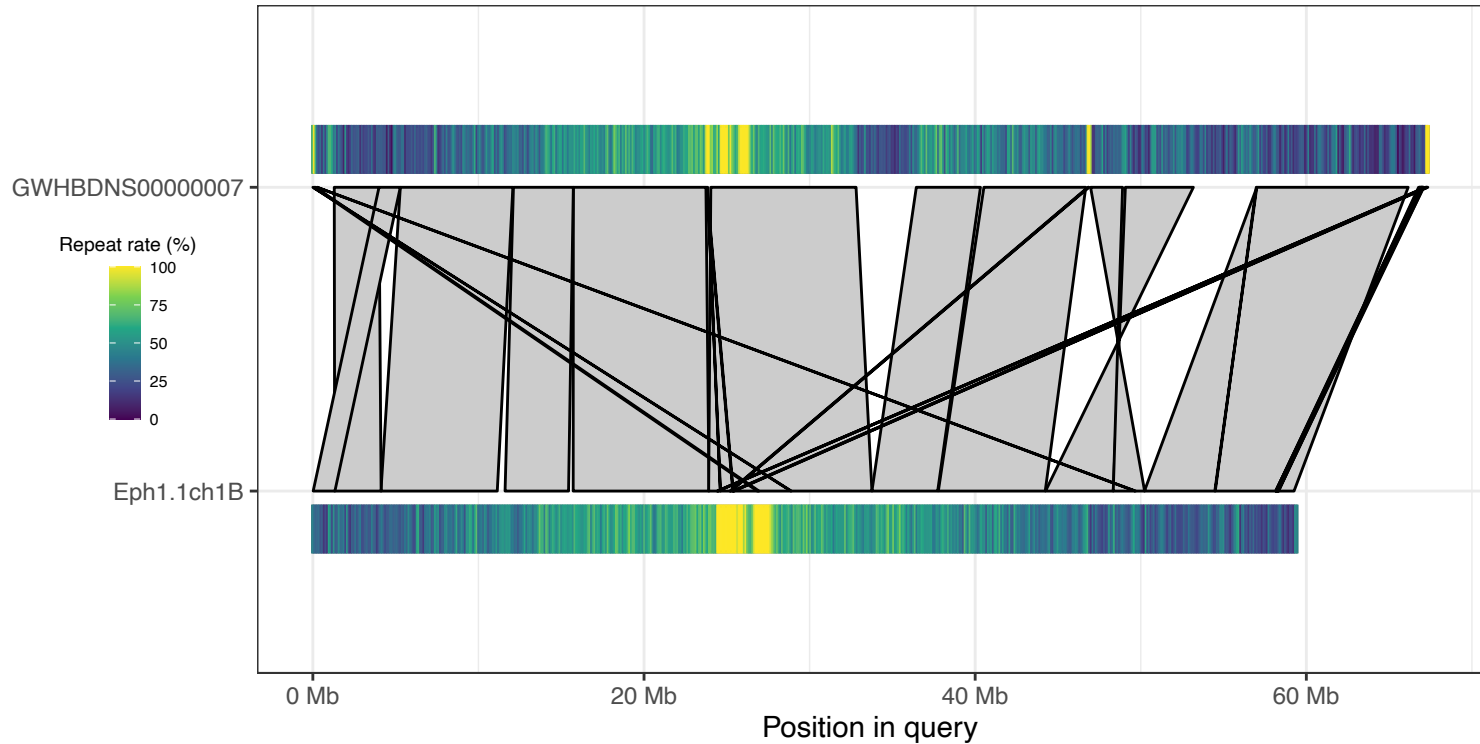

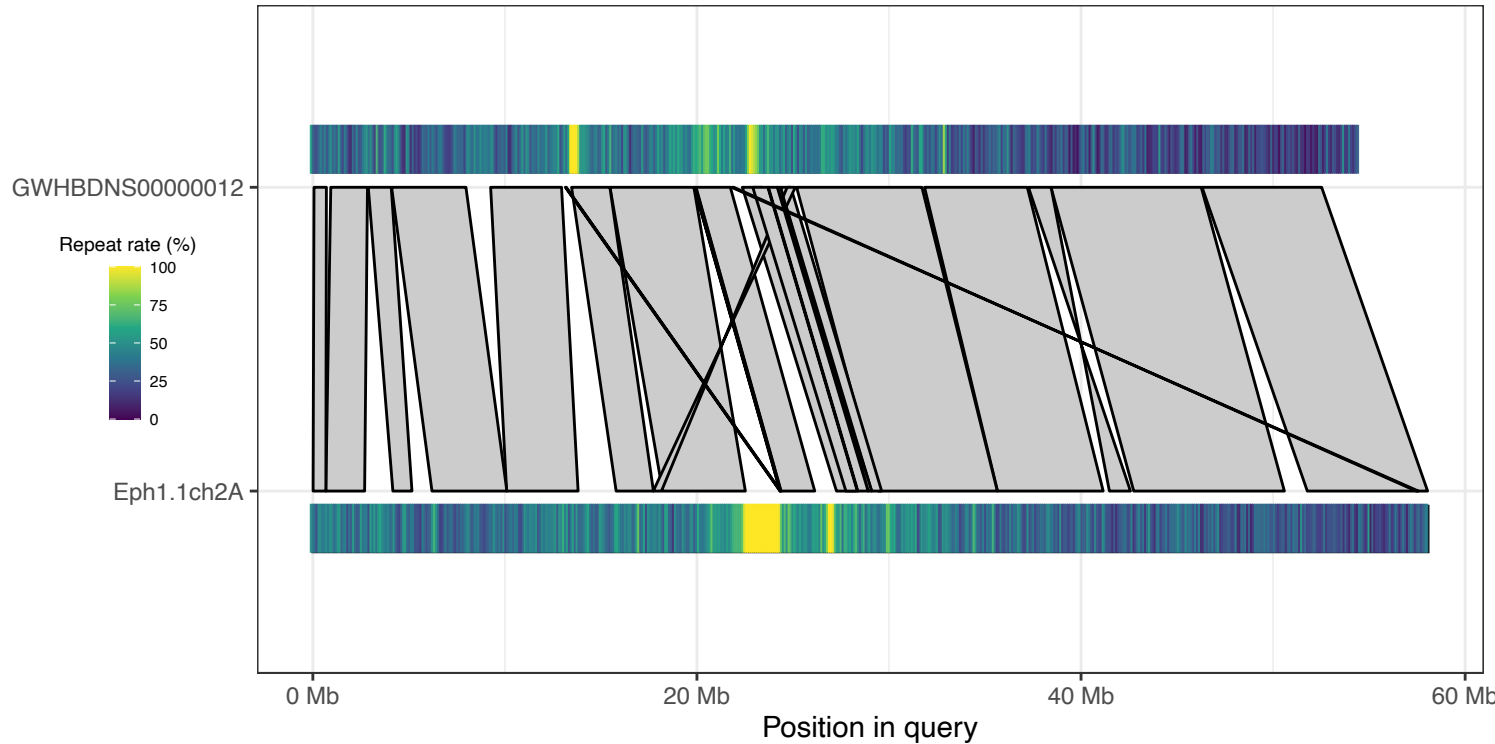

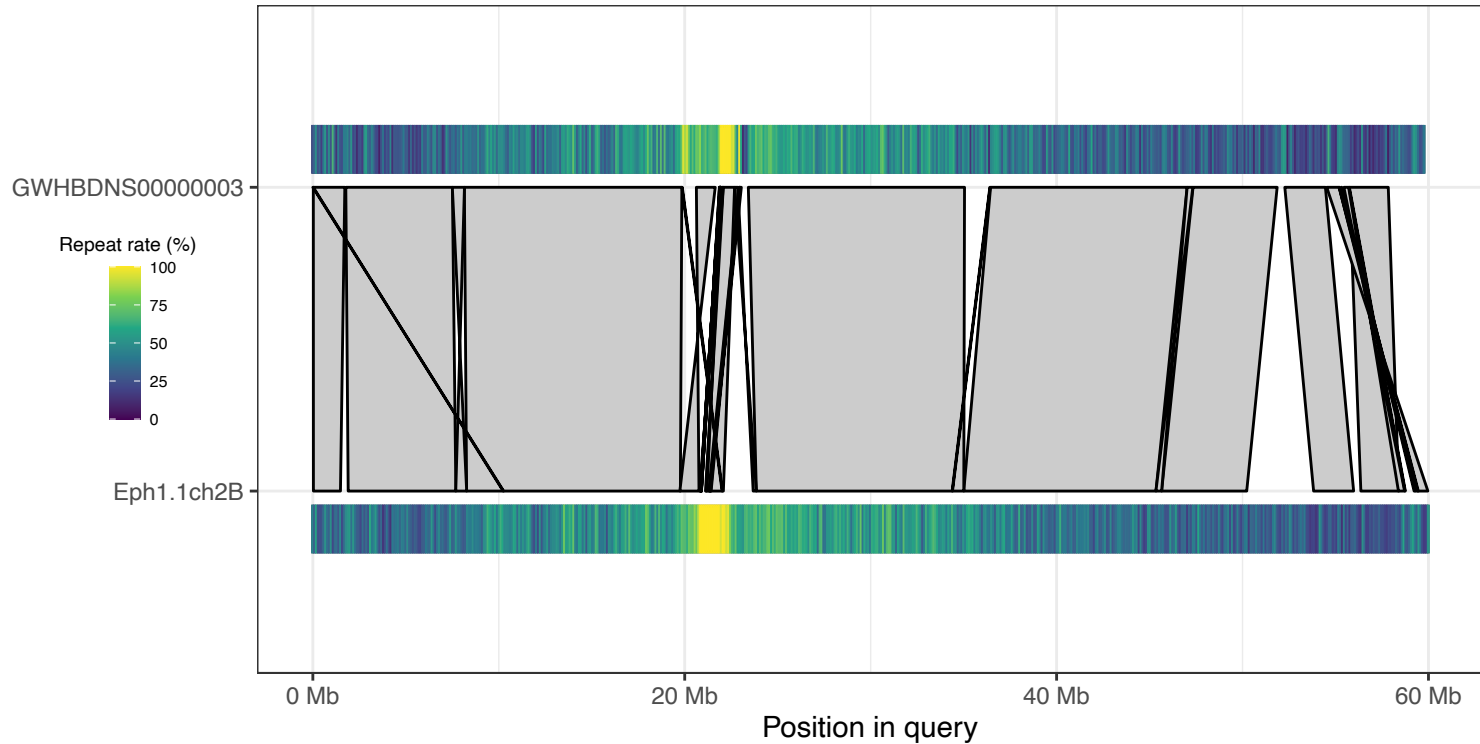

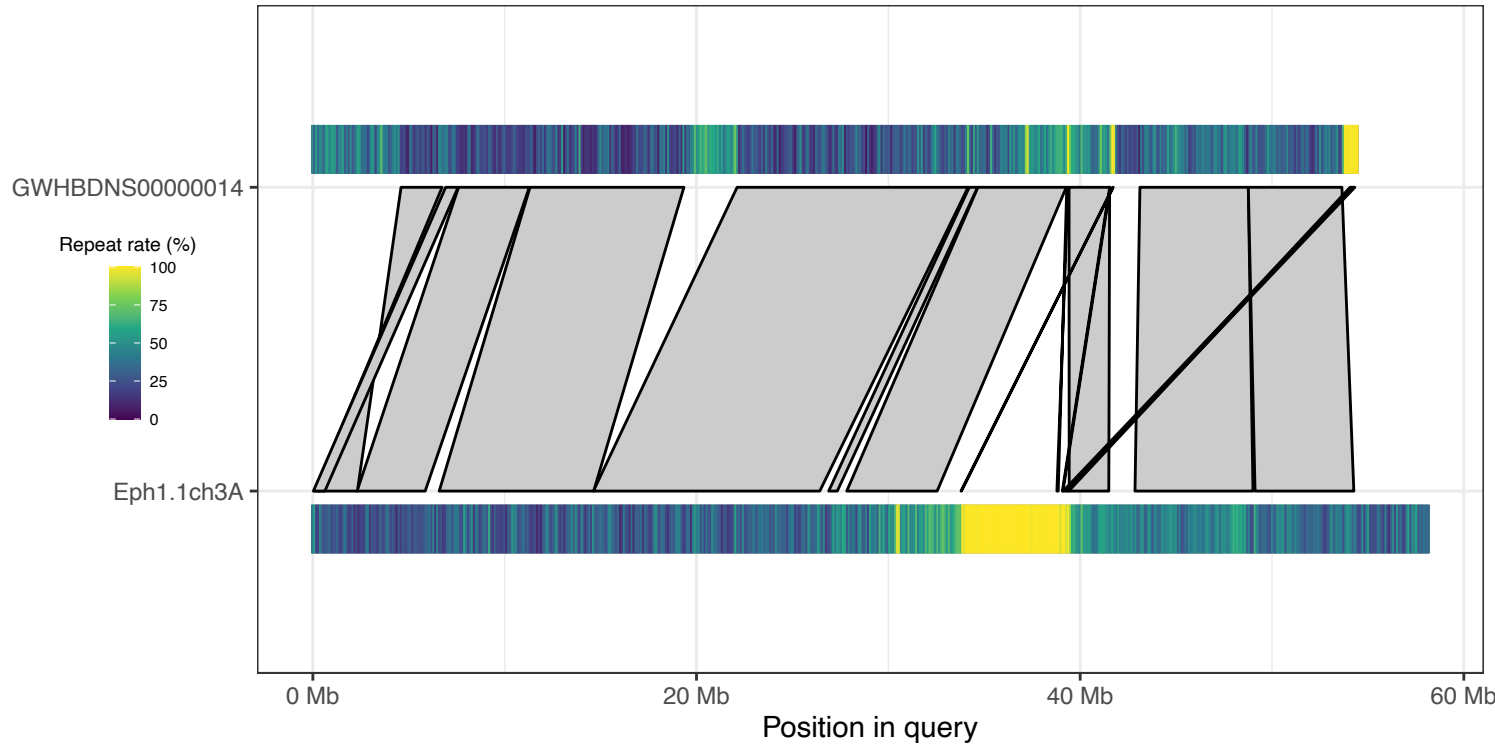

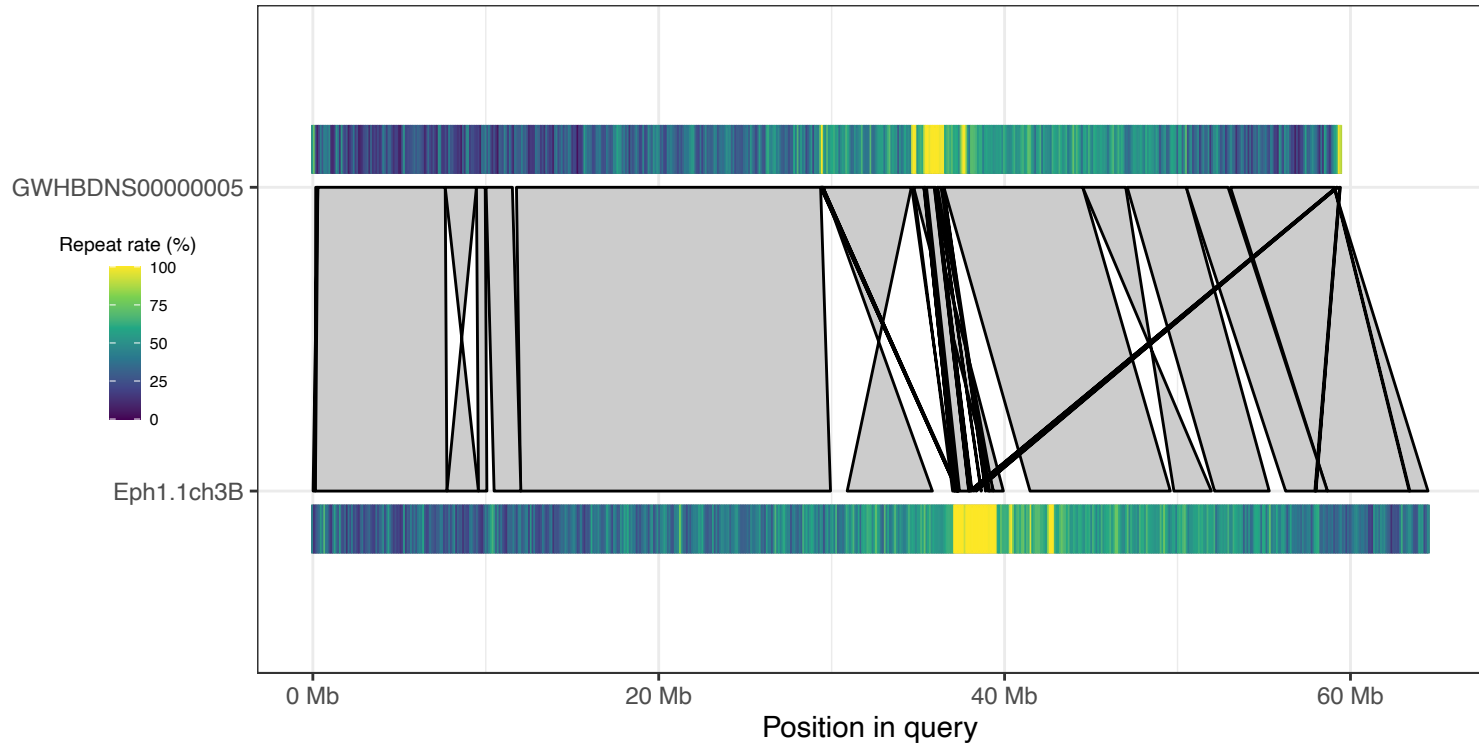

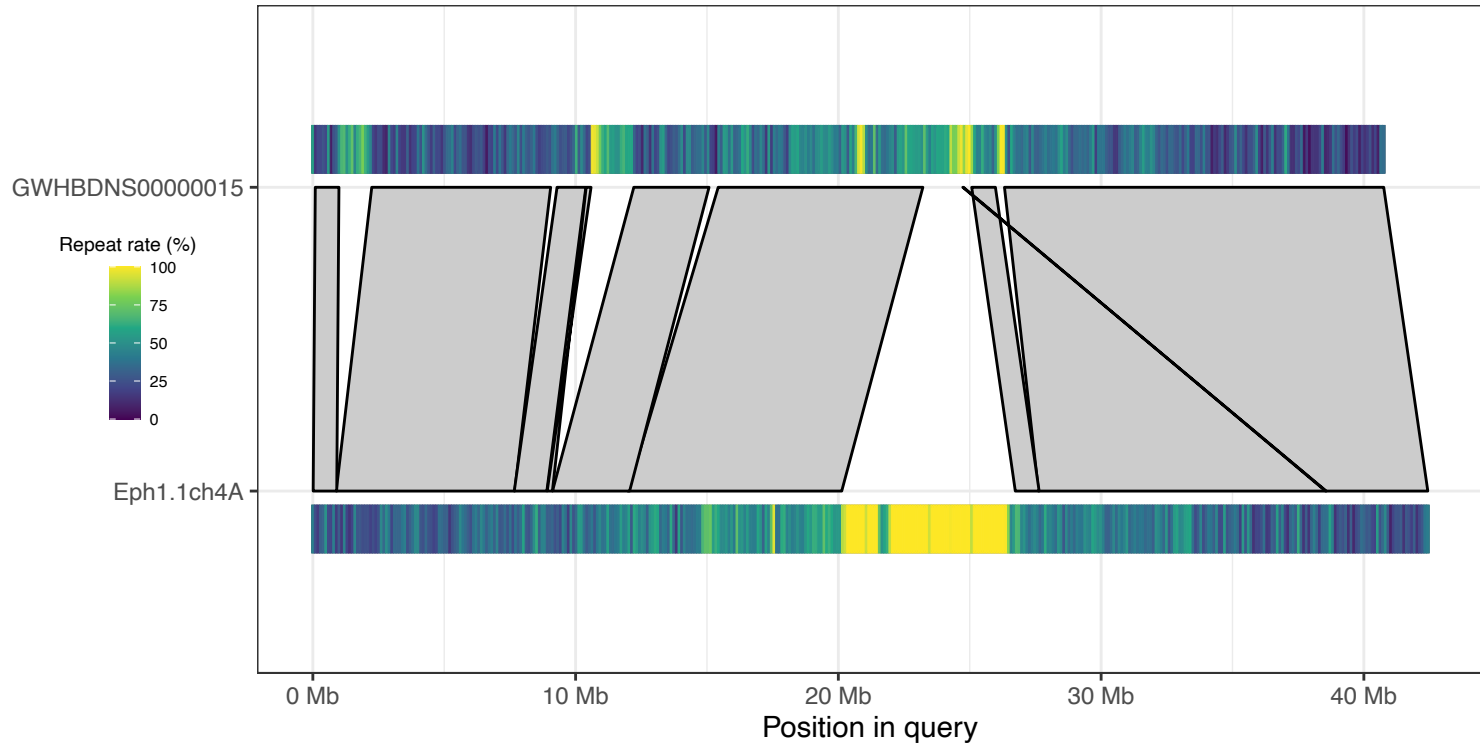

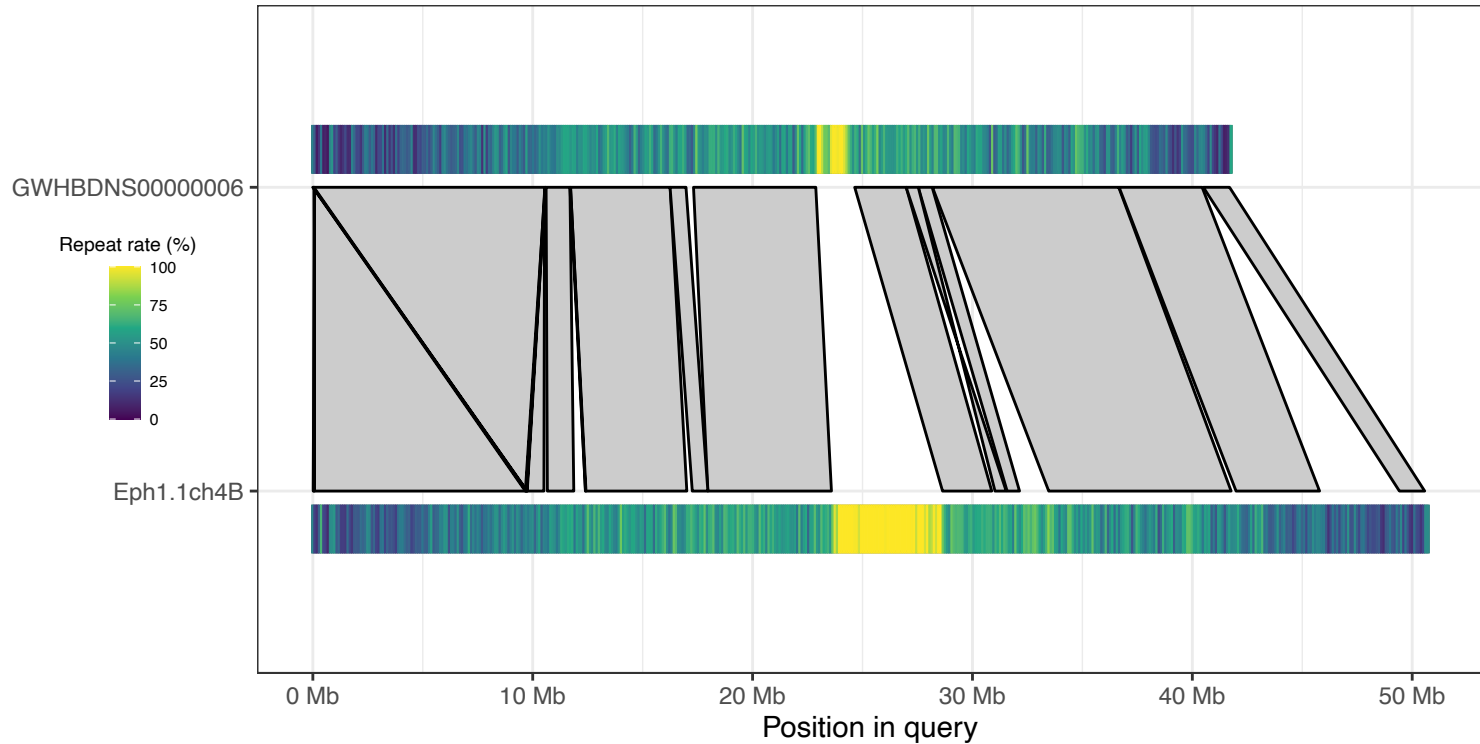

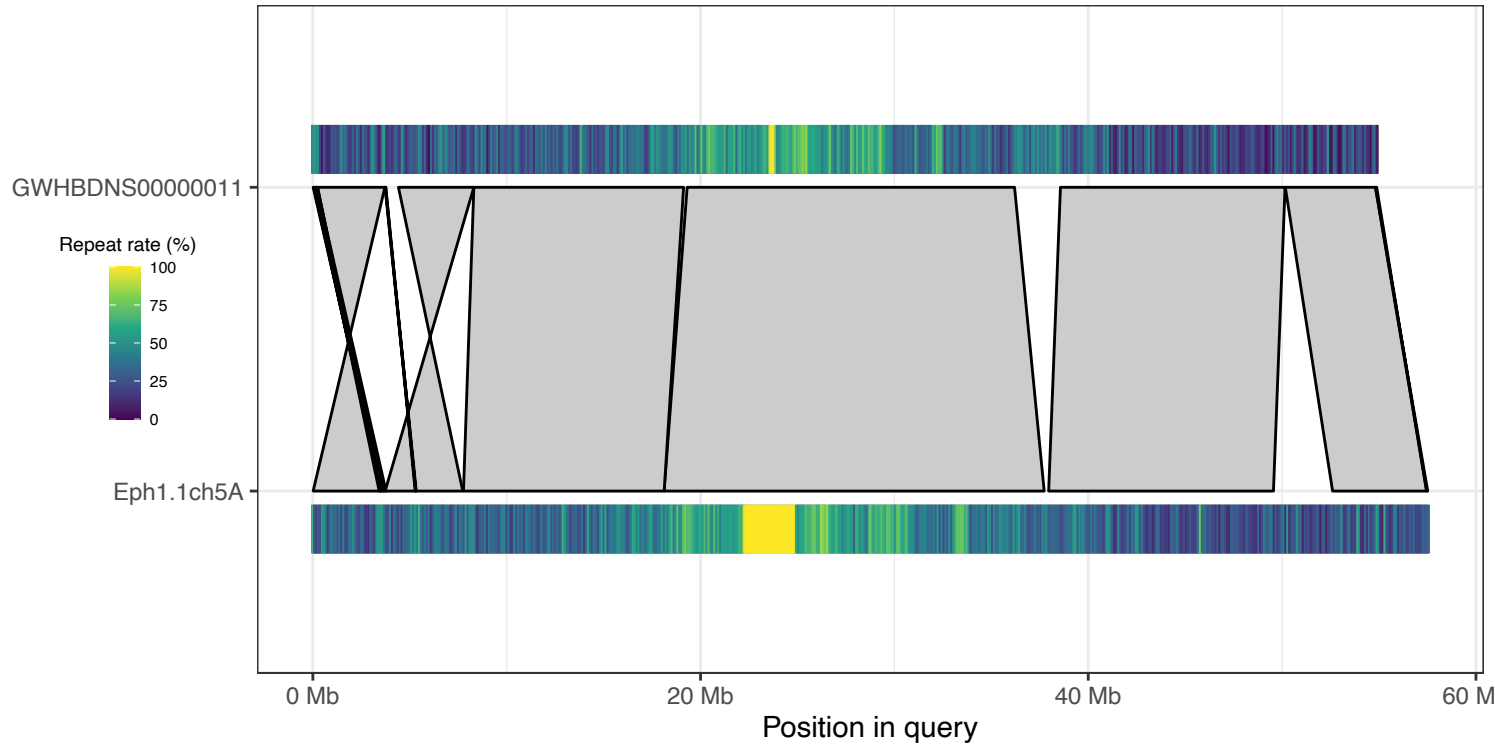

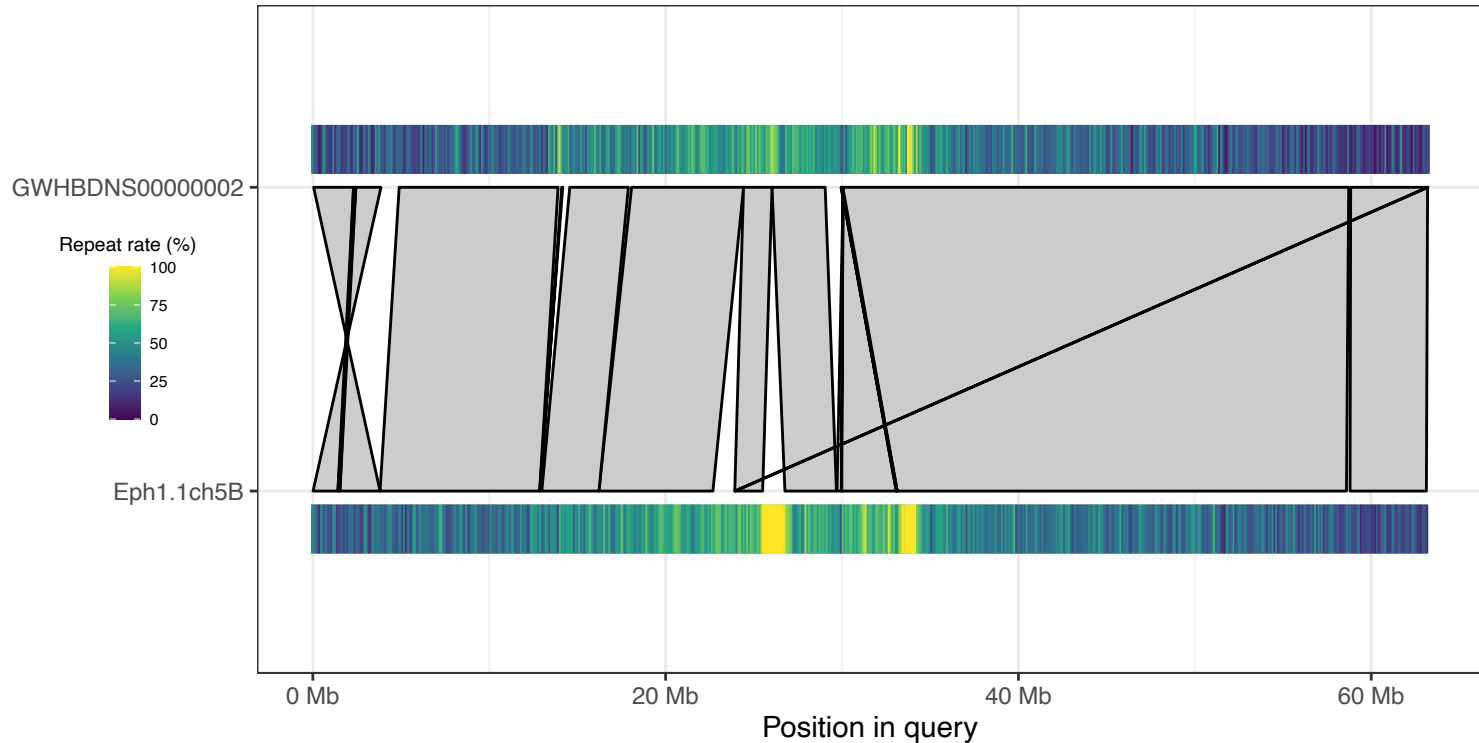

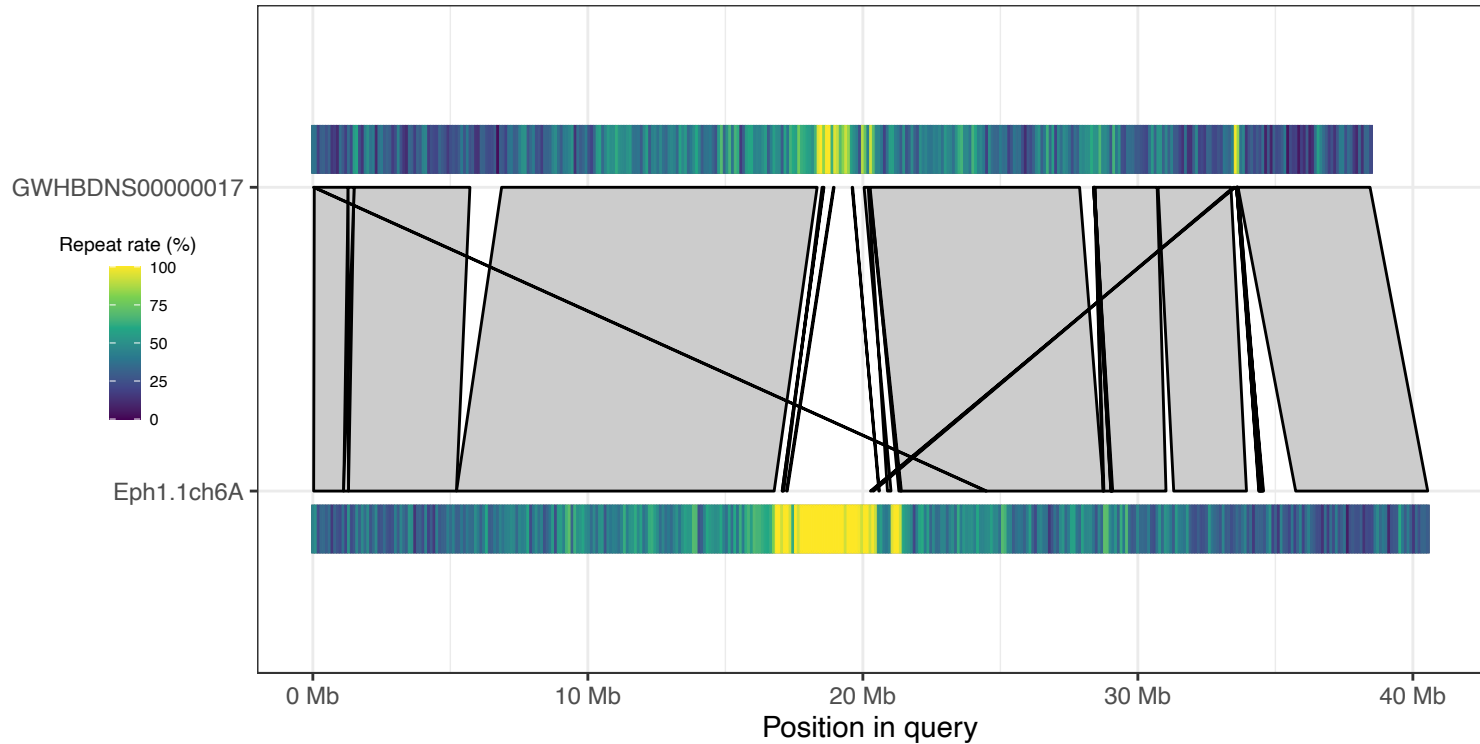

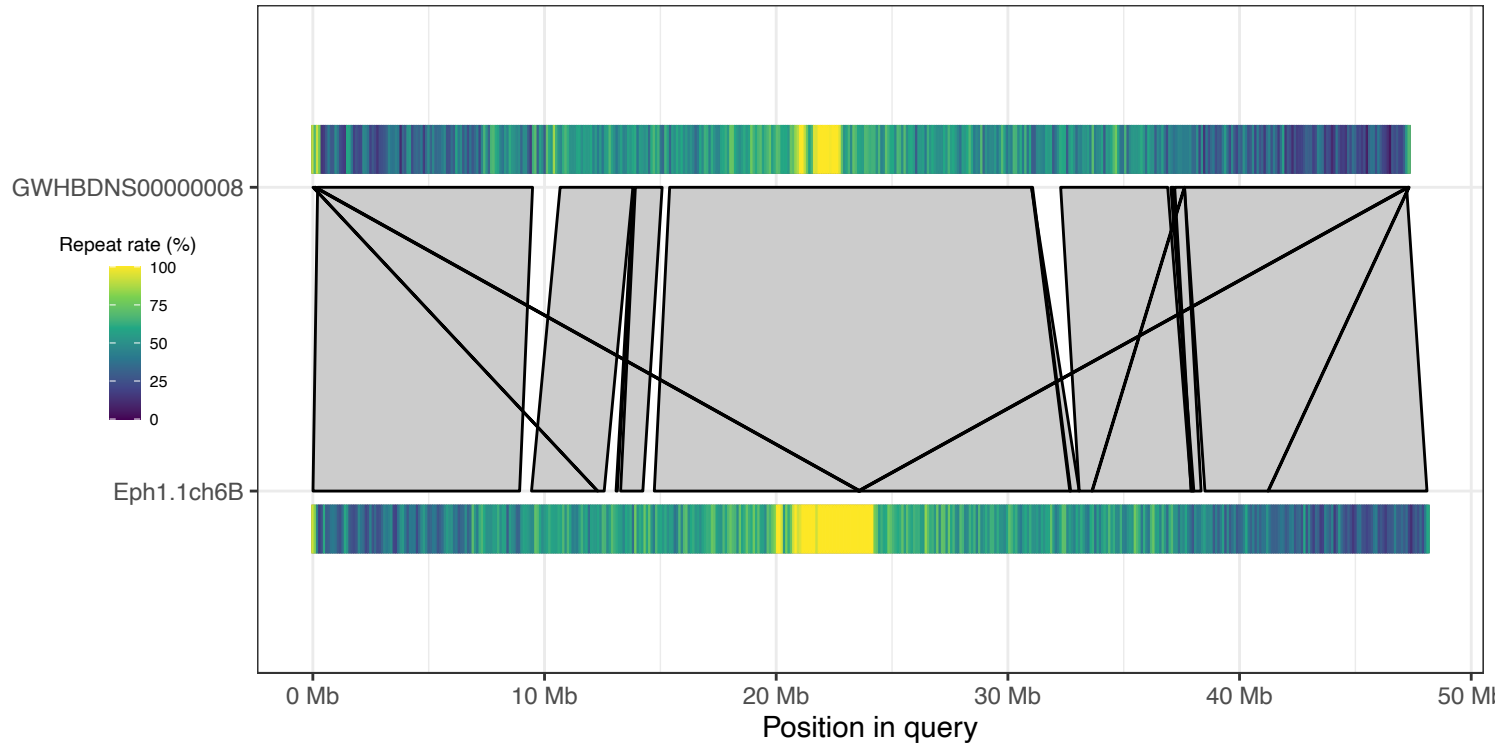

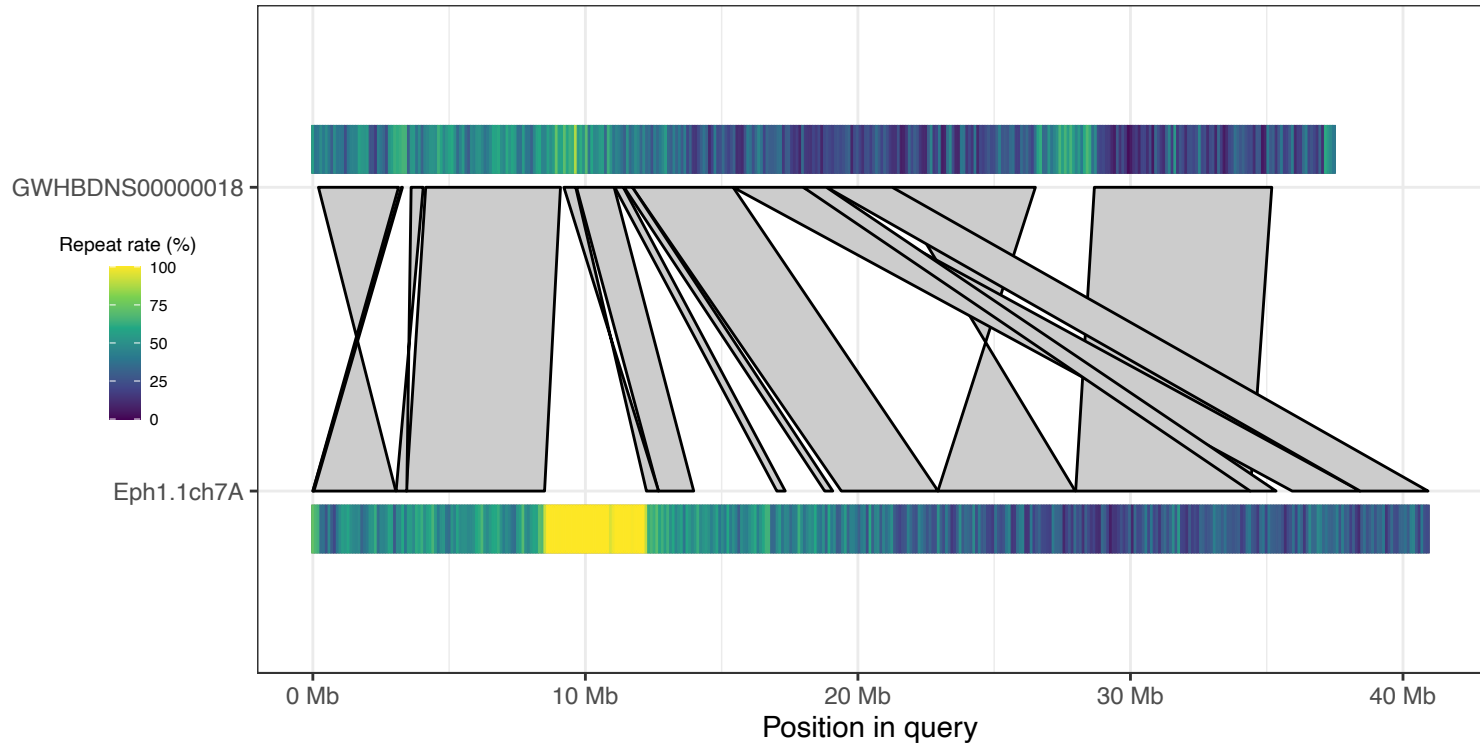

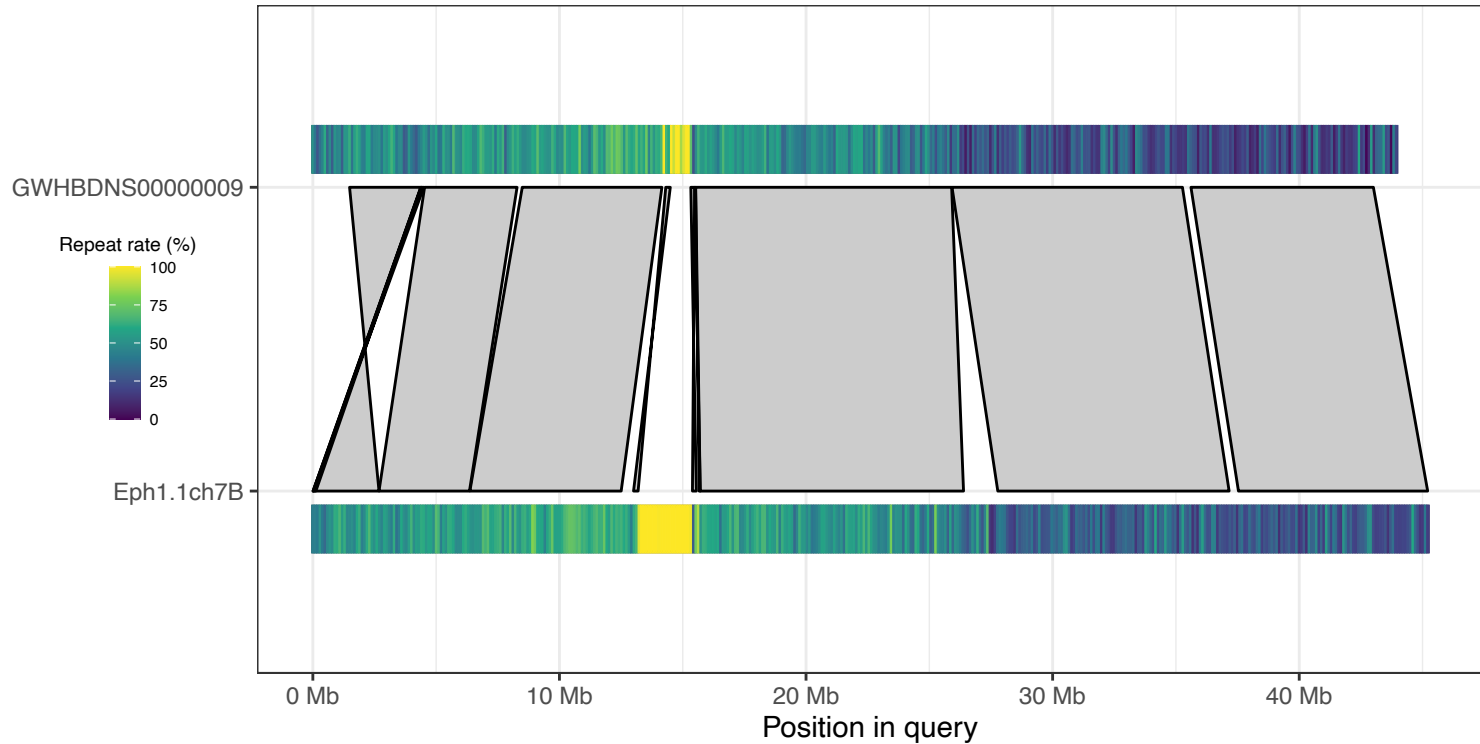

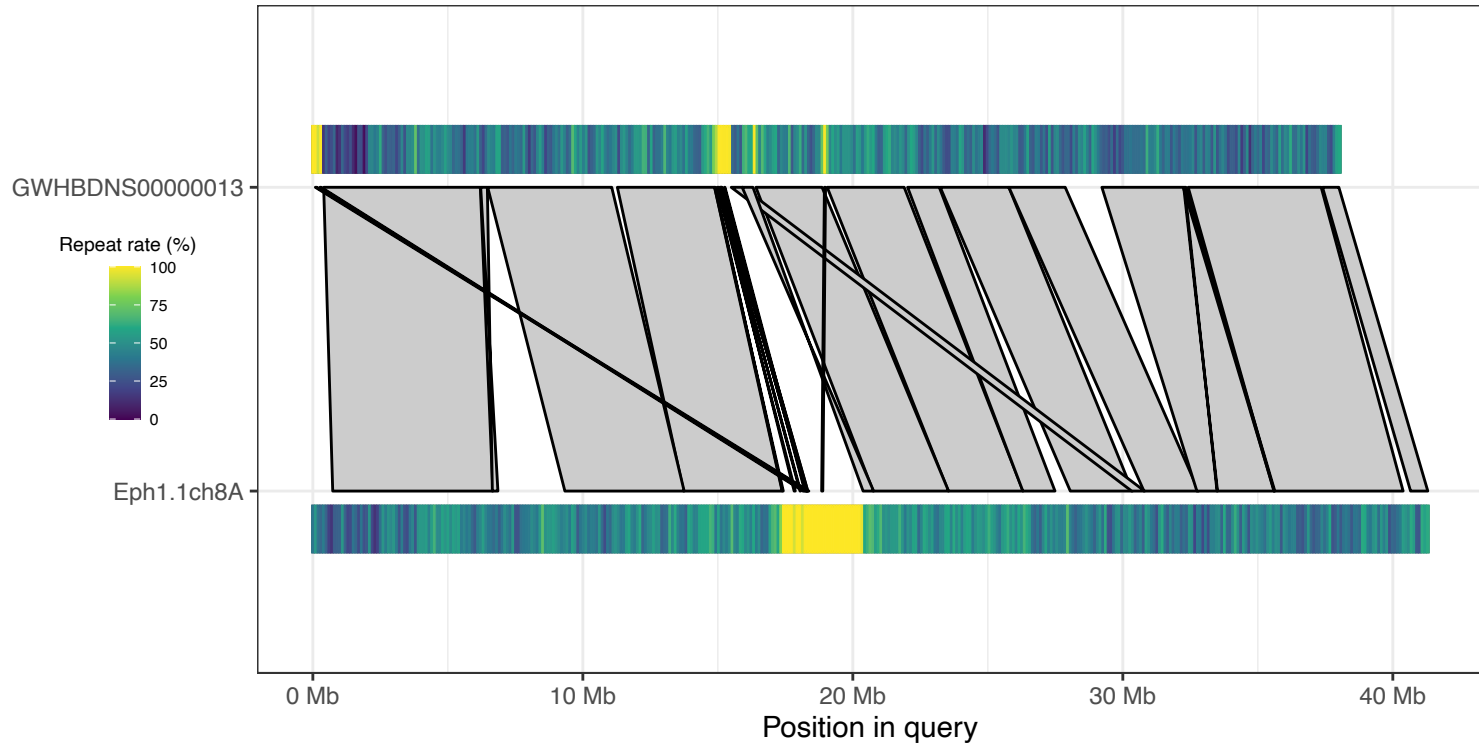

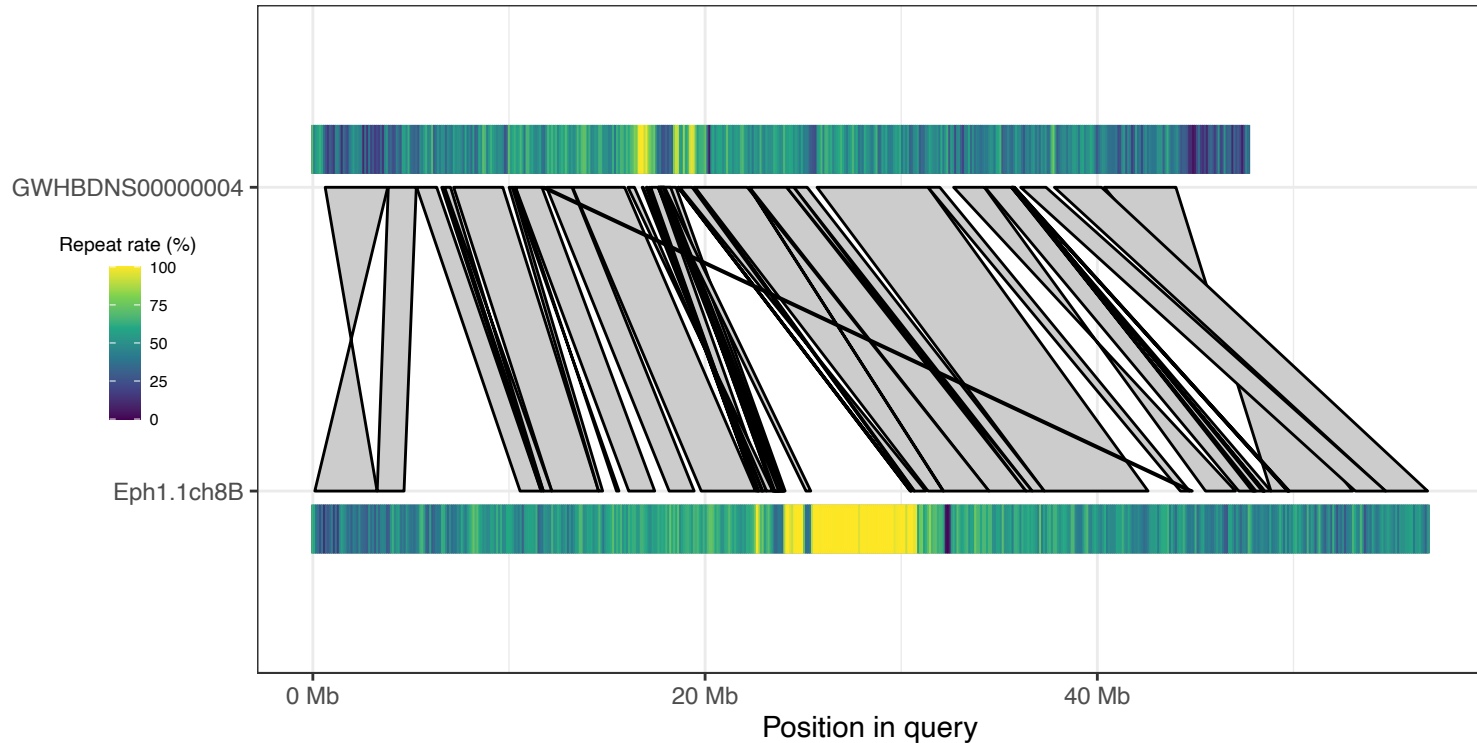

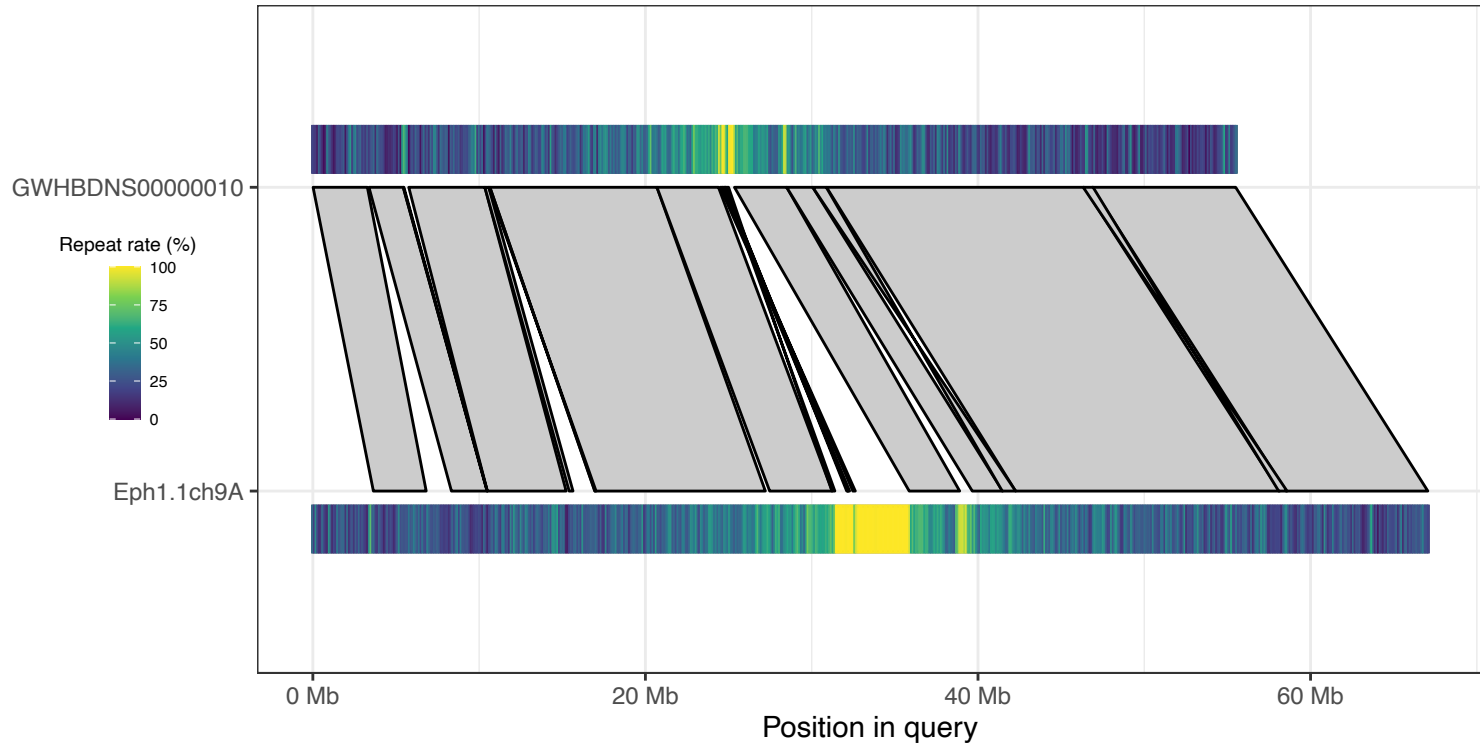

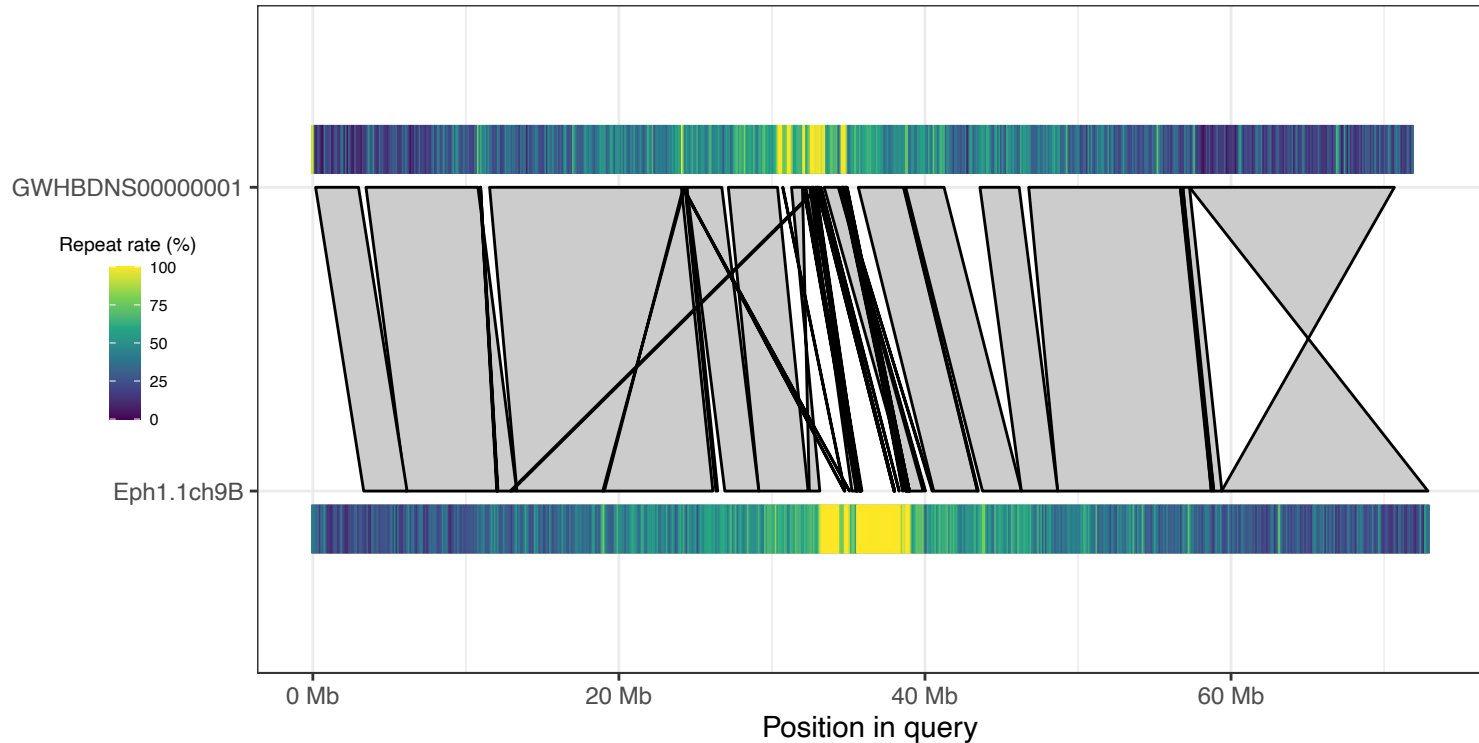

Supplement: dsad023_suppl_Supplementary_Figures_S4 [file dsad023_suppl_supplementary_figures_s4.pdf]

A

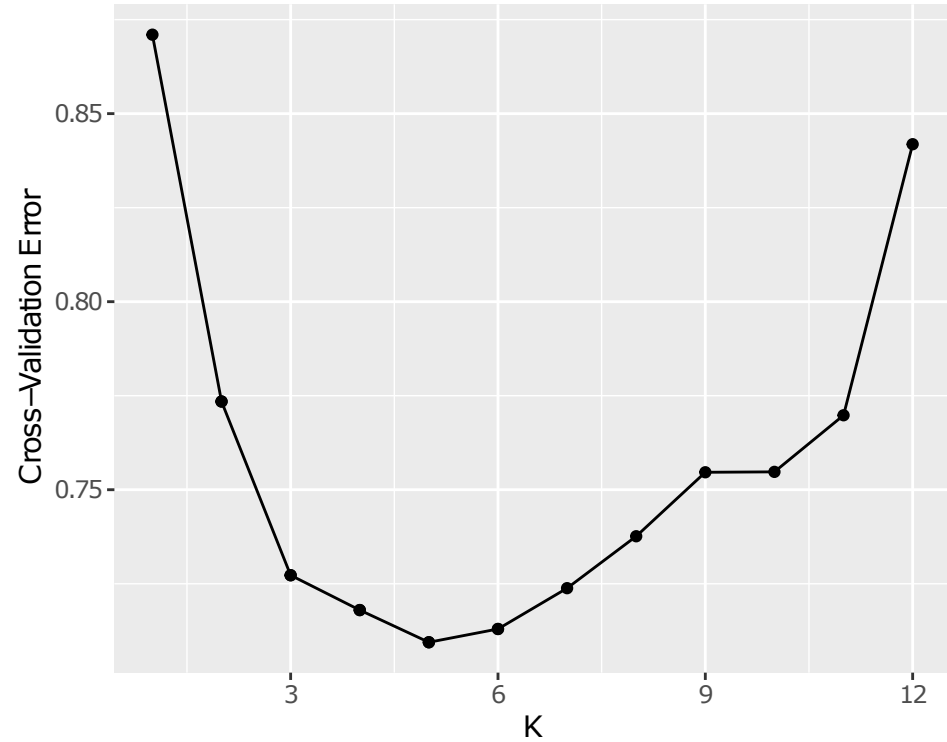

B

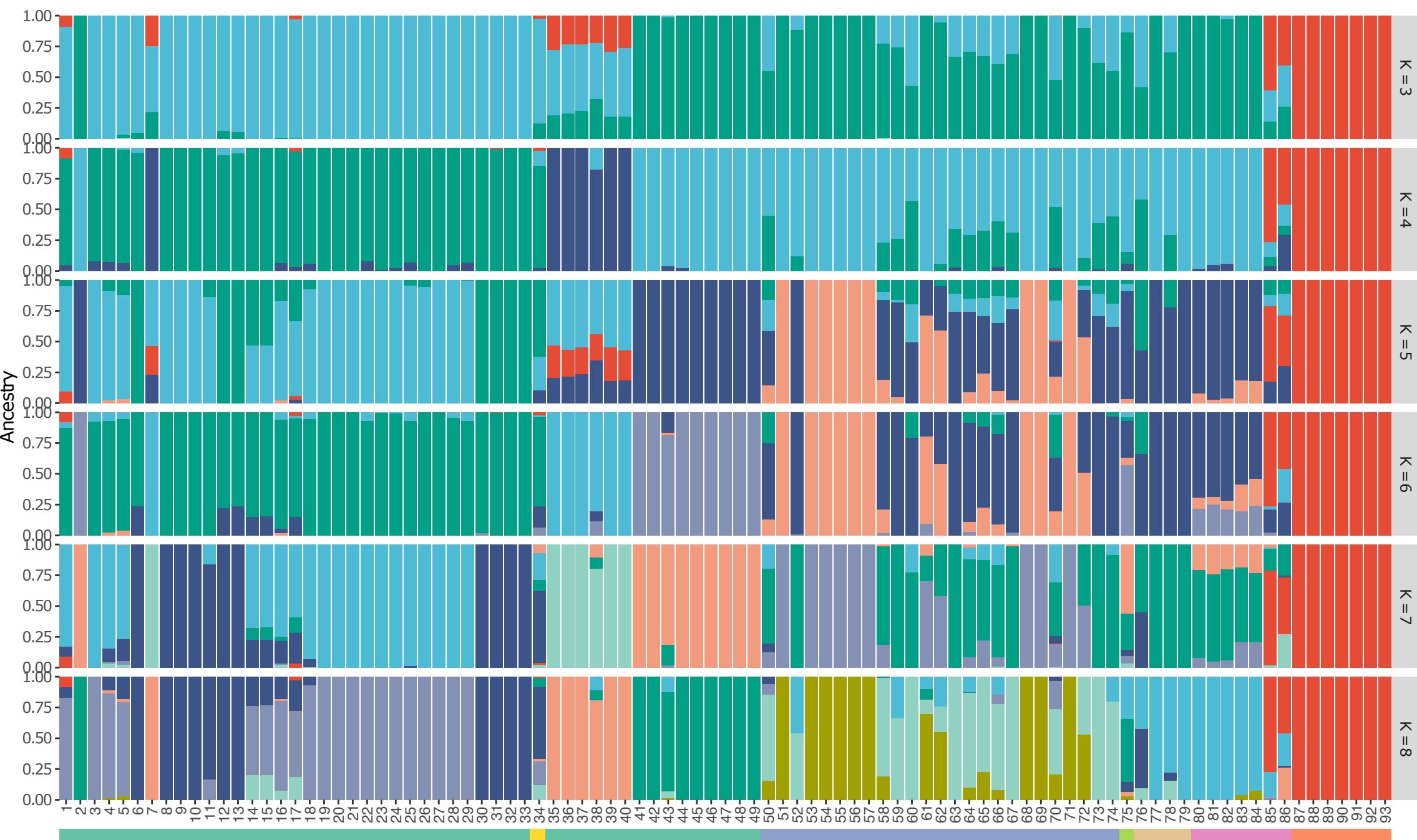

Supplement: dsad023_suppl_Supplementary_Figures_S5 [file dsad023_suppl_supplementary_figures_s5.pdf]
