# Supplementary material for: Telomere-to-telomere genome assembly of an allotetraploid pernicious weed, Echinochloa phyllopogon
Source: DNA Res. 2023 Nov 7;30(5):dsad023. doi: 10.1093/dnares/dsad023 (PMC10634394; doi:10.1093/dnares/dsad023)
Supplement: dsad023_suppl_Supplementary_Figure_Table_Legends [file dsad023_suppl_supplementary_figure_table_legends.docx]

**Supplementary Figure and Table Legends**

Supplementary figure 1. Dot plot comparing the alignment between EPH_r1.0, which includes all reads, and EPH_r0.1, which has been downsampled. Red lines indicate scaffolded contigs in EPH_r0.1.

Supplementary figure 2. Relationship between physical and genetic distance on the contig of EPH_r1.0. (A) Alignments and correlation between physical and genetic distance on six scaffolded contigs. (B) The marker position of contigs (EPH_r1.0).

Supplementary figure 3. Comparative analysis among *E. phyllopogon* (EPH_r1.1) and *E. hploclada*. (A) Comparison of the genome sequence and structure. Dots and colors indicate genome structure and sequence similarity, respectively. (B) Genetic distance of homologous chromosomes. *S. italica* (Sit) is outgroup. The blue letters of chromosome of *E. phyllopogon* indicate A genome and green indicate B genome.

Supplementary figure 4. Alignment region and repeat occupancy between *E. phyllopogon* EPH_r1.1 and eo_v2*.*

Supplementary figure 5. Population genetic structure analysis of *E. phyllopogon* worldwide. (A) Cross-valid error (B) K = 3–8.

Supplementary table 1. Detailed statistics of the genome assembly and gene prediction of *E. phyllopogon*.

Supplementary table 2. The number of reads and mapping rate of ddRAD-Seq.

Supplementary table 3. Linkage groups constructed by genetic mapping.

Supplementary table 4. Annotation list of high-confident genes.

Supplementary table 5. Subgenome-specific enriched gene list

Supplementary table 6. The number of reads and mapping rate of resequence worldwide.
